# Supplementary figures and images for: Mapping out Min protein patterns in fully confined fluidic chambers
Source: eLife. 2016 Nov 25;5:e19271. doi: 10.7554/eLife.19271 (PMC5217063; doi:10.7554/eLife.19271)

(a)

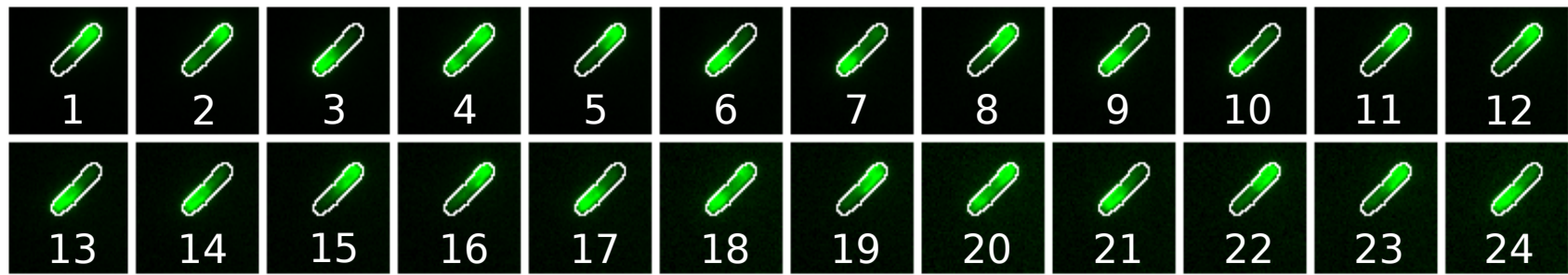

(b)

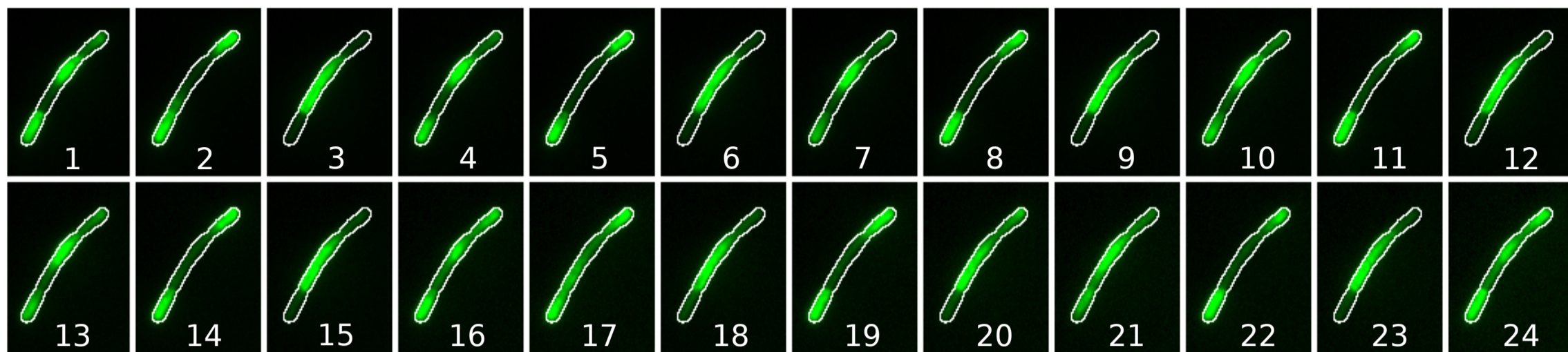

(c)

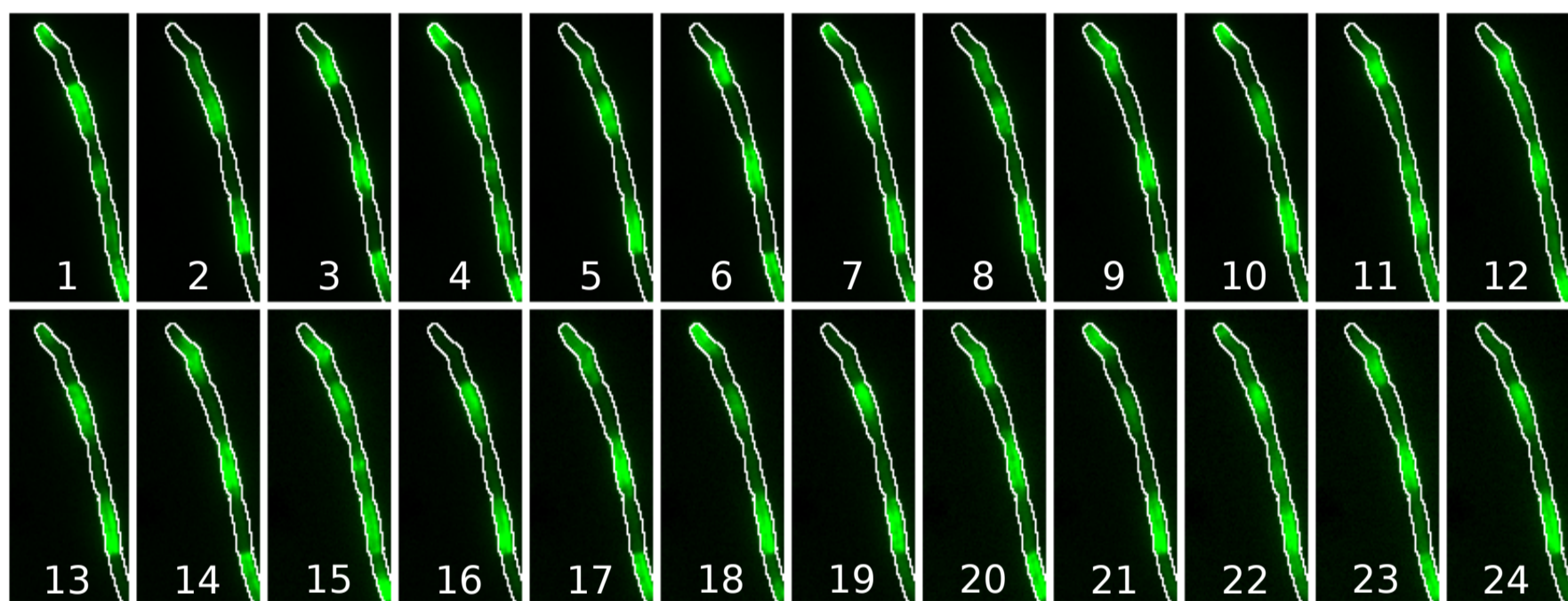

(d)

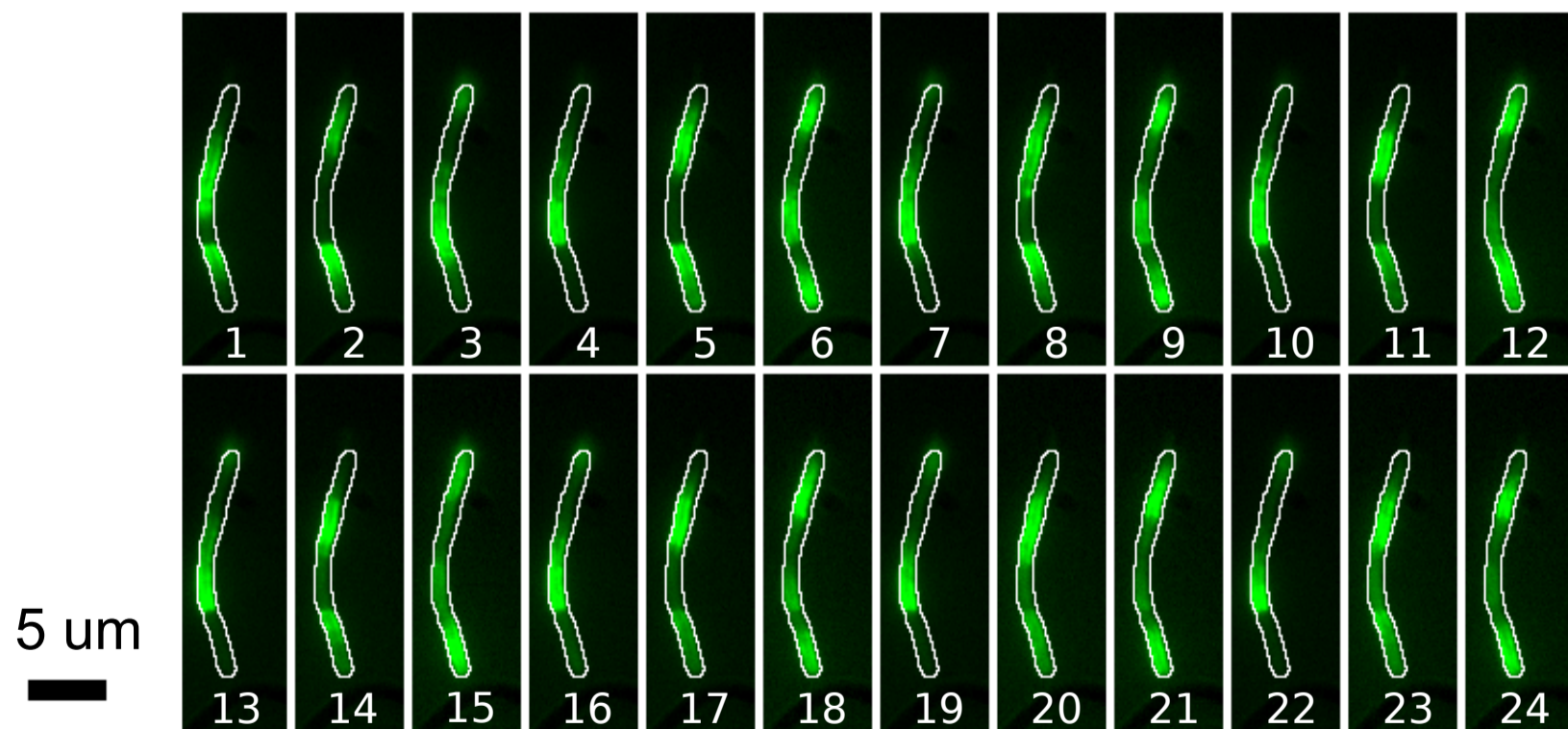

(e)

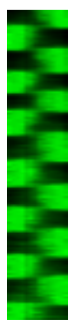

(f)

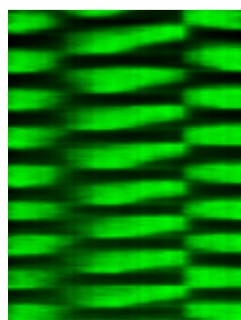

(g)

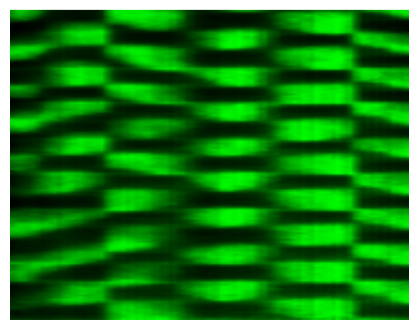

(h)

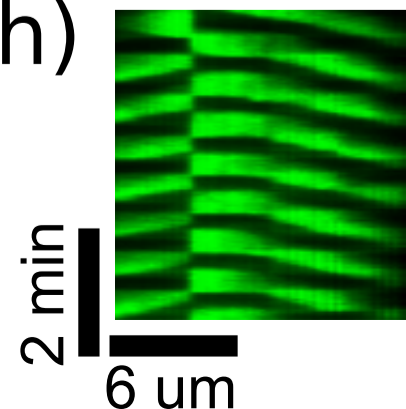

Supplement: Supplementary file 1. — (a–d) Four examples of in vivo MinD oscillations in cells with various length of strain FW1919 (see Wu et al., 2016) Mol Sys Biol 12: 873). Panels show montages of the corresponding supporting Supplementary file 2. Time difference between frames is 12 s. Scale bar (5 μm) is shown to the left of panel (d). Cell boundaries are marked in white. (a) Pole-to-pole oscillations in a wild type cell of normal length (3.8 μm). (b) A triple-node oscillations in a Cephalexin-division-inhibited cell with a length of 11.9 μm. (c) Multiple-node oscillations in a Cephalexin-division-inhibited cell (19.9 μm). (d) Aberrant triple-node oscillations in a Cephalexin-division-inhibited cell (14.4 μm). For the last case, instead of the regular pattern, where triple-point-oscillations occur between a middle zone and the two poles (see panels (b and f)), the middle zone seems to originate in the center of the cell and while traveling toward one pole, a MinD zone is established on the opposite pole. (e–h) Corresponding kymographs of the MinD intensity along the cells’ length for panels (a–d) respectively. Scale bar for the kymographs are shown next to panel (h). DOI: http://dx.doi.org/10.7554/eLife.19271.020 [file elife-19271-supp1.pdf]
